# Sex Differences in Clinical Features of Early, Treated Parkinson’s Disease

**DOI:** 10.1371/journal.pone.0133002

**Published:** 2015-07-14

**Authors:** Erika F. Augustine, Adriana Pérez, Rohit Dhall, Chizoba C. Umeh, Aleksandar Videnovic, Franca Cambi, Anne-Marie A. Wills, Jordan J. Elm, Richard M. Zweig, Lisa M. Shulman, Martha A. Nance, Jacquelyn Bainbridge, Oksana Suchowersky

**Affiliations:** 1 Department of Neurology, University of Rochester Medical Center, Rochester, NY, United States of America; 2 The University of Texas School of Public Health, Austin, TX, United States of America; 3 Barrow Neurological Institute, Phoenix, AZ, United States of America; 4 Department of Neurology, Brigham and Women’s Hospital and Harvard Medical School, Boston, MA, United States of America; 5 Neurological Clinical Research Institute, Massachusetts General Hospital, Boston, MA, United States of America; 6 Department of Neurology, University of Kentucky, Lexington, KY, United States of America; 7 Department of Neurology, Massachusetts General Hospital and Harvard Medical School, Boston, MA, United States of America; 8 Department of Public Health Sciences, Medical University of South Carolina, Charleston, SC, United States of America; 9 Louisiana State University Health Sciences Center, Shreveport, LA, United States of America; 10 Department of Neurology, University of Maryland School of Medicine, Baltimore, MD, United States of America; 11 Struthers Parkinson’s Center, Minneapolis, MN, United States of America; 12 University of Colorado Skaggs School of Pharmacy and Pharmaceutical Sciences, Aurora, CO, United States of America; 13 Department of Medicine, University of Alberta‎, Edmonton, AB, Canada; 14 Department of Medical Genetics, University of Alberta‎, Edmonton, AB, Canada; 15 Department of Pediatrics, University of Alberta‎, Edmonton, AB, Canada; CSIR-Central Drug Research Institute, INDIA

## Abstract

**Introduction:**

To improve our understanding of sex differences in the clinical characteristics of Parkinson’s Disease, we sought to examine differences in the clinical features and disease severity of men and women with early treated Parkinson’s Disease (PD) enrolled in a large-scale clinical trial.

**Methods:**

Analysis was performed of baseline data from the National Institutes of Health Exploratory Trials in Parkinson’s Disease (NET-PD) Long-term Study-1, a randomized, multi-center, double-blind, placebo-controlled study of 10 grams of oral creatine/day in individuals with early, treated PD. We compared mean age at symptom onset, age at PD diagnosis, and age at randomization between men and women using t-test statistics. Sex differences in clinical features were evaluated, including: symptoms at diagnosis (motor) and symptoms at randomization (motor, non-motor, and daily functioning).

**Results:**

1,741 participants were enrolled (62.5% male). No differences were detected in mean age at PD onset, age at PD diagnosis, age at randomization, motor symptoms, or daily functioning between men and women. Differences in non-motor symptoms were observed, with women demonstrating better performance compared to men on SCOPA-COG (Z = 5.064, p<0.0001) and Symbol Digit Modality measures (Z = 5.221, p<0.0001).

**Conclusions:**

Overall, men and women did not demonstrate differences in clinical motor features early in the course of PD. However, the differences observed in non-motor cognitive symptoms suggests further assessment of the influence of sex on non-motor symptoms in later stages of PD is warranted.

## Introduction

Sex-specific differences are often manifesting in disease risk, clinical manifestations, clinical management, and prognosis in widely varied disorders ranging from cardiovascular disease to neurodegenerative disorders.[1–6] Underlying factors associated with these disparities include: differences in disease biology, differing pharmacokinetics and pharmacodynamics, as well as variations in provider treatment practice. Recommendations for more systematic evaluation of sex differences in health in both pre-clinical and clinical research, and greater participation in clinical trials by women, have been endorsed by several professional organizations and government agencies.[7–9]

Although Parkinson’s Disease (PD) is a common disorder with aging[10], sex differences are not well understood. The incidence and prevalence of disease are greater in men, who carry an approximate 1.5–2 times greater risk for development of PD compared to women.[11–15] While there is consensus regarding disparate risk of developing PD, there are conflicting data regarding sex differences in the timing of symptom onset and the nature of clinical manifestations. Sex differences in clinical manifestations may indicate important mechanistic factors with respect to neurobiology, therapeutic development, disease prevention and clinical management.[16–18] In this analysis of participants in a large scale clinical trial, we sought to improve current knowledge of sex differences in the clinical phenotype of early, treated PD.

## Methods

We analyzed baseline data from the National Institutes of Health Exploratory Trials in Parkinson’s Disease (NET-PD) Long-term Study-1 (LS-1), a randomized, multi-center, double-blind, placebo-controlled study of 10 grams of oral creatine/day in individuals who were within 5 years of PD diagnosis and who were on dopaminergic therapy for fewer than two years (early treated PD). Between March 2007 and May 2010, 1,741 subjects from 45 United States and Canadian sites were enrolled. Details of the trial design, baseline sample characteristics, and the primary trial results have been published.[19, 20]

We sought to evaluate whether there are sex differences in age milestones related to disease presentation and trial randomization, including: age at symptom onset, age at PD diagnosis, age at randomization, years since symptom onset, years since PD diagnosis, and length of time between symptom onset and diagnosis. We also evaluated differences in specific clinical features: retrospective patient-reported symptoms at the time of diagnosis (motor symptoms) and symptoms present at the time of randomization (motor symptoms, non-motor symptoms, and daily functioning).

### Statistical methods

Normality of the data was tested using the Kolmogorov Smirnov test. Differences in proportions for categorical variables (demographic characteristics) between men and women were evaluated using the chi-square test or the Fisher exact test depending on assumptions. Age at symptom onset, age at PD diagnosis, age at randomization, years since symptom onset, years since PD diagnosis, and length of time between symptom onset and diagnosis were analyzed for differences in demographic characteristics between men and women; adjustment for multiple comparisons was made via Bonferroni correction with a type I error level of 0.05/6 = 0.0083. Disease presentation and randomization age milestones were analyzed using a series of t-tests for unequal variances. We compared men and women on their time from symptom onset to PD diagnosis using the Wilcoxon rank sum test because this variable was not normally distributed.

We evaluated three outcomes related to clinical features: 1. Motor symptoms at the time of diagnosis and at the time of trial randomization, 2. non-motor symptoms at the time of trial randomization, and 3. daily functioning at the time of trial randomization. Motor symptoms at diagnosis were defined by: resting tremor, rigidity, bradykinesia, postural instability, and other symptoms. To evaluate differences between men and women in motor symptoms at randomization, we compared five variables (ambulatory capacity, UPDRS motor score, the percent of the waking day that dyskinesias are present, the presence of early morning dystonia, and the percent of the waking day that the subject is "Off" on average) between men and women. Ambulatory capacity identifies the sum of the response to the following questions administered in the UPDRS: (i) falling, (ii) freezing, (iii) walking, (iv) gait and (v) postural stability. From the UPDRS part IV (Complications of therapy), we assessed the proportion of the waking day that dyskinesias were present with three categories of response: none, 1–25% of the day and >25% of the day. Due to small sample size in some categories, we collapsed the categories of 1–25% and >25% of the day into present (some part of the day) versus absent (none). We assessed the proportion of the waking day that the subject is "Off" on average with five categories of response: none, 1–25% of the day, 26–50% of the day, 51–75% of the day and 76–100% of the day. Again, due to small sample size in some categories, we collapsed the categories of 26–50%, 51–75% and 76–100% into one category of >25% of the day.

We compared non-motor symptoms at the time of trial randomization between men and women using six non-motor variables: UPDRS Part I (Mentation) score, Scale for Outcome of Parkinson Disease Cognition (SCOPA-COG), Symbol Digit Modalities (SDM), Beck Depression Inventory II total score (BDI), self-reported symptomatic orthostasis, and self-reported sleep disturbance.

We compared daily functioning at the time of randomization between men and women using seven global functioning variables: UPDRS Part II (ADL) score, Schwab and England ADL (S&E ADL), total functional capacity (TFC), Parkinson’s Disease Questionnaire (PDQ-39) summary index, EuroQoL 5-D (EQ5D) utility score, Modified Rankin Scale, and care level.

Twenty three variables related to clinical features were tested for differences between men and women; therefore adjustment for multiple comparisons was made via Bonferroni correction with a type I error level of 0.05/23 = 0.002.

We also evaluated differences between men and women in motor and non-motor symptoms at randomization and daily functioning at the time of randomization after adjustment for age, marital status, duration of PD, and levodopa equivalent daily dose at randomization using: (i) linear regression (for ambulatory capacity, UPDRS Part I–Mentation, UPDRS Part II–ADL, UPDRS Part III—Motor, SCOPA-COG, SDM, BDI, S&E ADL, TFC, PDQ-39 Summary Index and EQ5D; (ii) logistic regression (for percent of the waking day that dyskinesias were present, presence of early morning dystonia, self-reported symptomatic orthostasis, self-reported sleep disturbance, and care level); and (iii) multinomial logistic regression (for the percent of the waking day that the subject was "Off" on average, using none as the reference category and for Modified Rankin Scale using “No significant disability despite symptoms” as the reference category).

### Ethics Statement

The NET-PD LS-1 trial was registered at www.clinicaltrials.gov (NCT00449865). Study procedures were reviewed and approved by the institutional review board for each site, prior to enrollment of participants, who each provided written informed consent to study participation. Participating sites are listed in the Appendix.

## Results

Of the 1,741 total participants, 1,123 were male (62.5%). Men were more likely than women to be married at randomization (Chi-square statistic = 67.8, p<0.0001). Other demographic characteristics were not different between men and women (Table 1). We did not observe differences between men and women in mean age at PD symptom onset (t = -1.27, df = 1186.3, p = 0.20), age at PD diagnosis (t = -1.37, df = 1191.7, p = 0.17), or age at randomization (t = -1.38, df = 1194.3, p = 0.16). The length of time between PD symptom onset and PD diagnosis was a mean 1.7 years for both men (SD 1.7) and women (SD 2.0) (Wilcoxon Rank sum test = -0.44, df = 1472.2, p-value = 0.66). Men and women were enrolled into the study on average 3.2 years (SD 2.0) and 3.3 years (SD 2.3) after symptom onset, respectively (t-test for unequal variance = -0.29, df = 1453, p-value = 0.77). Randomization occurred a mean of 1.5 years (SD 1.1) after PD diagnosis for both sexes (t-test = 0.2, df = 1739, p-value = 0.84).

**Table 1:** Demographic Characteristics (N = 1,741).

	Female		Male	
	n	%	n	%
Age (years)				
<30	1	0.2	0	0
30–40	9	1.5	13	1.2
40–50	73	11.8	98	8.7
50–60	174	28.2	307	27.3
60–70	228	36.9	456	40.6
70–80	118	19.1	225	20.0
≥80	15	2.4	24	2.1
Non-Hispanic whites	559	90.5	1012	90.1
Education				
<High school	33	5.3	50	4.5
High school/GED	96	15.5	127	11.3
Some college/associate	181	29.3	236	21.0
Bachelors	163	26.4	314	28.0
Graduate/professional	145	23.5	396	35.3
Marital Status				
Never Married	37	6.0	55	4.9
Now Married	433	70.1	959	85.4
Widowed/Divorced/Separated	148	23.9	109	9.7
Care level				
Chronic care/Full-time skilled nursing	7	1.1	12	1.1
Home	611	98.9	1111	98.9
Current employment activities				
1.Working Full Time	174	28.2	495	44.1
2.Retired	231	37.4	427	38.0
3.Working part-time	98	15.9	134	11.9
4.Not working, on disability pay	31	5.0	41	3.7
5.Homemaker	61	9.9	0	0.0
6.Unemployed and looking for work	9	1.5	15	1.3
7.Other	11	1.8	11	1.0
8.Student	2	0.3	0	0.0

There were no differences in the proportion of men and women demonstrating any cardinal features of PD (resting tremor, rigidity, bradykinesia, postural instability, or “other symptoms”) at the time of diagnosis (Table 2). The three most common “other symptoms” reported as being present at the time of diagnosis included: gait disturbance (n = 68), micrographia or handwriting change (n = 38), and hypophonia or voice change (n = 29). Additional symptoms reported less frequently included: drooling or swallowing dysfunction, hypomimia, dystonia, fine motor incoordination, non-rest tremor, anosmia, sensory disturbance, weakness, stooped posture, muscle cramping, problems with memory, pain, mood change, fatigue, constipation, sleep disturbance, freezing, and falls. There were no differences between men and women in motor symptom frequency or severity at the time of randomization (Table 2), including after controlling for age, marital status, duration and amount of levodopa equivalence daily dose at randomization.

**Table 2:** Motor symptoms ^‡^.

	Female		Male		p-value		Sig
At the time of diagnosis of Parkinson Disease	n	%	n	%			
Resting Tremor					†	0.024	No
Yes	511	82.8	877	78.2			
No	106	17.2	244	21.8			
Rigidity					†	0.651	No
Yes	522	86.6	962	87.4			
No	81	13.4	139	12.6			
Bradykinesia					†	0.854	No
Yes	566	92.2	1021	91.8			
No	48	7.8	91	8.2			
Postural instability					†	0.201	No
Yes	127	20.8	202	18.3			
No	483	79.2	903	81.7			
Other motor symptoms					†	0.595	No
Yes	110	17.9	188	16.9			
No	503	82.1	925	83.1			
At the time of trial randomization							
** **	n	Mean(SD)	n	Mean(SD)			
Ambulatory Capacity score**	616	1.8 (1.6)	1123	1.6 (1.5)	¥	0.014	No
UPDRS motor	615	17.0 (8.6)	1118	18.2 (8.2)	*	0.003	No
	n	%	n	%			
Individuals with dyskinesias					†	0.021	No
Some part of the day	31	5.0	31	2.8			
None of the day	587	95.0	1091	97.2			
Individuals with early morning dystonia					†	0.003	No
Yes	106	17.2	133	11.9			
No	512	82.9	989	88.2			
Individuals with "Off" periods					+	0.819	No
More than 25% of the day	18	2.9	39	3.48			
1–25% of the day	119	19.3	215	19.16			
No “Off” periods	481	77.8	868	77.36			

Sig: Significant after Bonferroni correction on the type I error level = 0.05/23 = 0.002.

† Two-sided Fisher exact test.

+ Chi square statistic = 0.399 (degrees of freedom = 2).

¥ t-test for unequal variances = 2.47 (Degrees of freedom = 1174.3).

* t-test for equal variances = -2.97 (Degrees of freedom = 1731).

‡ Differences between number of observed values and 1741 subjects is due to missing values for these variables.

** Sum of the response to the following UPDRS questions: (i) falling, (ii) freezing, (iii) walking, (iv) gait and (v) postural stability.

In terms of non-motor features, women demonstrated better SCOPA-COG and better SDM performance compared to men (Table 3). Initial analysis of daily functioning revealed better UPDRS ADL performance for women compared to men (Table 4). After controlling for age, marital status, duration and amount of levodopa equivalence daily dose at randomization, differences in SCOPA-COG total score (p<0.0001) and SDM (p<0.0001) remained significant. However, this did not hold true for differences in UPDRS ADL (p = 0.003).

**Table 3:** Non-motor symptoms at the time of trial randomization^‡^.

	Female		Male		Test Statistic		p-value	Sig
	n	Mean (SD)	n	Mean (SD)				
UPDRS Mentation score	618	1.3 (1.4)	1123	1.3 (1.4)	†	-0.567	0.571	No
SCOPA-COG total score	615	31.1 (5.5)	1116	29.8 (5.2)	†	5.064	<0.0001	Yes
Symbol Digit Modalities (total correct responses)	617	46.4 (12.0)	1119	43.3 (11.4)	†	5.221	<0.0001	Yes
Beck Depression Inventory total score	615	7.1 (5.7)	1121	6.8 (5.5)	†	0.941	0.347	No
	n	%	n	%				
Individuals with sleep disturbance (e.g. insomnia or hypersomnolence)					+		0.795	No
Yes	228	36.9	407	36.3				
No	390	63.1	715	63.7				
Individuals with symptomatic orthostasis					+		0.662	No
Yes	81	13.1	156	13.9				
No	537	86.9	966	86.1				

† Wilcoxon two sample test statistic two sided normal approximation.

+ Two-sided Fisher exact test.

‡ Differences between observed values and 1741 subjects is due to missing values for these variables.

Sig: Significant adjusting for Bonferroni correction on the type I error level = 0.05/23 = 0.002.

**Table 4:** Daily functioning features at the time of trial randomization^‡^.

	Female		Male		Test Statistic		p-value	Sig
	n	Mean (SD)	n	Mean (SD)				
UPDRS ADL score	617	6.8 (4.1)	1123	7.4 (3.9)	†	-3.523	0.0004	Yes £
S&E ADL score	618	91.0 (6.9)	1122	91.1 (6.8)	†	-0.012	0.990	No
TFC	617	11.9 (1.6)	1122	12.1 (1.3)	†	-0.822	0.411	No
PDQ-39 Summary Index score	617	14.0 (10.9)	1121	12.8 (10.5)	†	2.268	0.023	No
EuroQol (generic instrument) EQ5D utility score	618	0.8 (0.2)	1123	0.8 (0.2)	†	-1.992	0.046	No
	n	%	n	%	** **	** **	** **	** **
Modified Rankin Scale score					+		0.026	No
1. No Symptoms at all	14	2.3	9	0.8				
2. No significant disability despite symptoms	459	74.3	885	78.8				
3. Slight disability	134	21.7	211	18.8				
4. Moderate Disability	11	1.8	18	1.6				
Care level					¥		1.000	No
Chronic care/Full-time skilled nursing	7	1.1	12	1.1				
Home	611	98.9	1111	98.9				

† Wilcoxon two sample test statistic two sided normal approximation.

+ Chi square statistic = 9.289 (degrees of freedom = 3).

¥ Two-sided Fisher exact test.

‡ Differences between observed values and 1741 subjects is due to missing values for these variables.

Sig: Significant adjusting for Bonferroni correction on the type I error level = 0.05/23 = 0.002.

£: Not significant after controlling for age, marital status, duration and amount of levodopa equivalent daily dose at randomization: p = 0.003.

## Discussion

PD is expected to affect more than 9 million individuals worldwide by 2030.[21, 22] This progressive neurodegenerative disorder significantly impacts quality of life and results in high societal economic burden.[22] Understanding sex-specific features of presentation, symptomatology, response to treatment, functional impact, and disease burden represents one critical aspect to improving care delivery to a growing population. Improved knowledge of sex-related differences will be valuable for clinical trial planning and assessment of treatment response.

Underlying the concept of sex-related differences, it has been hypothesized that endogenous and exogenous estrogen exposure may be one of the factors involved in neuroprotection or individual symptomatic effects in Parkinson’s disease.[23, 24] The MPTP (1-methyl-4-phenyl-1,2,3,6-tetrahydropyridine) mouse model of PD demonstrates a sex-based differential in nigrostriatal degeneration, with greater MPTP-induced toxicity observed in males. In addition, administration of 17β estradiol is protective against MPTP-induced toxicity in both sexes (mice). This positive effect is potentially mediated by estrogen receptors, expressed on both glial and neuronal cells.[25]

However, clinical studies of sex and PD are less clear regarding sex-related differences, reporting varied and sometimes conflicting results. For instance, some studies estimate age at symptom onset to be approximately two years later in women compared to men [11, 26], however, this has not been replicated in other studies [27–30]. Similarly, a delay in the timing of initial evaluation by a PD specialist for women compared to men has also been reported but not replicated.[31] In early PD, before treatment with levodopa or dopamine agonists, some studies show no difference in manifestation or severity of motor symptoms between men and women.[11, 26, 27] On the other hand, two studies suggest that women more often present with tremor-predominant PD.[26, 32], while other studies show higher UPDRS postural stability scores in females [27, 32], and greater rigidity in males [27]. There may be sex differences in non-motor symptoms as well: women report higher severity of cardiovascular, fatigue, and mood-related symptoms [19, 25] compared to men who report higher severity of sexual dysfunction [14], sialorrhea [14, 23, 24] and daytime sleepiness [14, 33]. At least three studies reporting on depression in PD indicate greater occurrence [27, 32, 34] and severity in women, while yet another study found no sex-related difference.[35] When examining dementia in PD, one study showed no sex differences in the prevalence of dementia,[36] while another study found greater development of dementia in men compared to women over a 10-year period [37]. Women have also been found to have higher Hoehn and Yahr stage [27, 38] and greater impairment in activities of daily living (ADLs) [27] at presentation. These conflicting results regarding potential sex differences in the epidemiology and clinical expression of PD require further clarification.

Our study of over 1700 individuals with early, treated PD did not find differences between men and women in age-based disease milestones or motor features. We previously reported a small, clinically-insignificant difference in motor symptoms between men and women in this cohort, however, this difference was not present following Bonferroni correction for multiple comparisons in the current analysis.[39]

There were, however, small-magnitude differences in non-motor symptoms. Women demonstrated better cognitive performance on two measures: the Symbol Digit Modalities Test (SDMT), a screening assessment for cognitive impairment, and the SCOPA-COG, a measure of memory and learning, attention, executive function, and visuo-spatial function. The absolute magnitude of difference in cognition scores between men and women was 1.3 for SCOPA-COG and 3.1 for SDMT, which may fall below the threshold of clinical significance. It is unclear whether the difference in cognitive performance represents evidence of a sex-specific effect of PD or represents general sex differences in the performance of cognitive tests. There are conflicting reports in the literature regarding the persistence of sex-specific differences in cognition with aging.[40, 41] The small difference in UPDRS ADL subscale outcomes was not found to be significant after controlling for age, marital status, and levodopa equivalent daily dose. Further, the small magnitude 0.6 point difference is less than what is typically considered a clinically meaningful difference.[42]

The varied results from previously published studies may relate to methodological differences in study design, such as prospective or retrospective data acquisition and differences in the study population. Sex differences may also vary throughout the course of PD. Of previously published studies, those with small samples were more likely to report sex differences in various clinical features; many of these findings equalized in larger samples. In our study of the largest clinical trial of PD patients to date, there were sex differences in non-motor manifestations, but we did not detect differences in disease onset, diagnosis, or motor symptoms. In our previous analysis, we showed that there was no difference in treatment between men and women in type of medications used or levodopa equivalent dosing.[39]

Our secondary analysis is strengthened by the large sample size and prospective acquisition of a broad battery of clinician-administered and self-report assessments, which allowed us to examine many domains of PD. The selected cognitive battery was intended to focus on cognitive deficits in PD, using a general screen (SDMT), as well as disease-specific assessments (UPDRS Part I Mentation and SCOPA-COG). However, this sample was drawn from a clinical trial designed to test the effects of a drug, not directly for epidemiological research. As a result, it is possible that our cohort of trial volunteers may not fully represent the general population of patients with PD.

The difference in cognition scores between men and women may represent an important finding. Increasing cognitive impairment correlates with overall disability, and PD with dementia is associated with lower quality of life and a higher degree of caregiver burden compared to PD without dementia.[43] Thus, this difference warrants further evaluation, especially if magnified later in the course of PD, at a time when greater disease burden would be anticipated. Further research is needed to elucidate potential differences in the long-term course of clinical signs and symptoms of PD in men and women, and to better delineate the significance of early sex-differences in non-motor symptoms.

### Appendix

Participating sites in the LS-1 study included: University of Alabama-Birmingham, University of South Florida, University of Southern California, Emory University School of Medicine, Oregon Health & Science University, University of Colorado, Johns Hopkins University, University of Texas Southwestern Medical Center, University of California San Francisco, University of Florida, Duke University, Louisiana State University Health Science Center-Shreveport, Michigan State University, Rush University Medical Center, University of Calgary, University of Pennsylvania, Beth Israel Deaconess Medical Center, Southern Illinois University, University of Michigan, Brigham and Women’s Hospital, University of Miami, Medical University of South Carolina, Pacific Health Research and Education Institute, University of Alberta, Washington University, University of Maryland School of Medicine, University of Vermont, Northwestern University, University of Kansas Medical Center, University of Kentucky, Dartmouth Hitchcock Medical Center, SUNY Downstate Medical Center, Thomas Jefferson University, Baylor College of Medicine, Georgia Health Sciences University, Institute for Neurodegenerative Disorders-New Haven, The Parkinson’s & Movement Disorder Institute-Fountain Valley, University of Virginia, Vanderbilt University Medical Center, Barrow Neurological Institute, UMDNJ Robert Wood Johnson Medical School, Malcolm Randall VA Medical Center, University of Florida-Jacksonville, Indiana University School of Medicine, and North Shore University Health System Research Institute.[19]
